# The relationship between diabetes and colorectal cancer prognosis: A meta-analysis based on the cohort studies

**DOI:** 10.1371/journal.pone.0176068

**Published:** 2017-04-19

**Authors:** Bo Zhu, Xiaomei Wu, Bo Wu, Dan Pei, Lu Zhang, Lixuan Wei

**Affiliations:** 1 Department of Cancer Prevention and Treatment, Cancer Hospital of China Medical University/ Liaoning Cancer Hospital & Institute, Dadong District, Shenyang, People’s Republic of China; 2 Department of Clinical Epidemiology and Evidence Medicine, The First Hospital of China Medical University, Heping District, Shenyang, People’s Republic of China; 3 Department of Anus and Intestine Surgery, The First Hospital of China Medical University, Heping District, Shenyang, People’s Republic of China; 4 Department of Occupational health, Liaohe Petrochemical Company of China National Petroleum Corporation, Xinglongtai District, Panjin, People’s Republic of China; National Cancer Center, JAPAN

## Abstract

**Introduction:**

Though a meta-analysis reported the effect of diabetes on colorectal prognosis in 2013, a series of large-scale long-term cohort studies has comprehensively reported the outcome effect estimates on the relationship between diabetes and colorectal prognosis, and their results were still consistent.

**Methods:**

We carried out an extensive search strategy in multiple databases and conducted a meta-analysis on the effect of diabetes on colorectal prognosis, based on the included 36 cohort studies, which contained 2,299,012 subjects. In order to collect more data, besides conventional methods, we used the professional software to extract survival data from the Kaplan-Meier curves, and analyzed both the 5-year survival rate and survival risk in overall survival, cancer-specific survival, cardiovascular disease—specific survival, disease-free survival, and recurrence-free survival, to comprehensively reflect the effect of diabetes on colorectal prognosis.

**Results:**

The results found that compared to patients without diabetes, patients with diabetes will have a 5-year shorter survival in colorectal, colon and rectal cancer, with a 18%, 19% and 16% decreased in overall survival respectively. We also found similar results in cancer-specific survival, cardiovascular disease—specific survival, disease-free survival, and recurrence-free survival, but not all these results were significant. We performed the subgroup analysis and sensitivity analysis to find the source of heterogeneity. Their results were similar to the overall results.

**Conclusions:**

Our meta-analysis suggested that diabetes had a negative effect on colorectal cancer in overall survival. More studies are still needed to confirm the relationship between diabetes and colorectal prognosis in cancer-specific survival, cardiovascular disease—specific survival, disease-free survival, and recurrence-free survival.

## Introduction

Colorectal cancer (CRC) is the third most commonly diagnosed cancer in global incidence and the fourth in mortality all over the world, and the incidence and mortality are higher in men than in women in most parts of the world [1]. In recent years, diagnosis and treatment had made a certain degree of progress, but CRC is still a very important public health problem in the world. Thus, early diagnosis, effective treatment and analysis prognosis were of great significance to reducing the CRC mortality. To guide decision-making for therapeutic strategies for CRC patients and improve their prognosis, a better understanding of the relevant factors affecting CRC prognosis is urgently needed.

Diabetes mellitus (DM) is one of the most common chronic and metabolism diseases. The number of people with DM worldwide has increased by two times in the past three decades[2]. An estimated 285 million people worldwide had diabetes mellitus in 2010, and the number of DM sufferers will rise to 439 million by 2030, represents 7.7% of the total adult population of the world aged 20–79 years[3]. The concurrence of DM pandemics with the growing burden of cancer globally has generated interest in defining the epidemiological and biological relationships between these medical conditions[3, 4].

DM can seriously affect quality of life. DM can not only cause neurological and vascular complications, but is also closely related to the occurrence, development and prognosis of cancer. Currently, more and more clinicians are considering whether patients have suffered from diabetes during the treatment of cancer, and diabetologists often have to manage diabetes in patients who are being treated for cancer[4]. Insulin resistance or compensatory hyperinsulinemia leads to hormonal and metabolic alterations, and is involved in the formation of the microenvironment for tumorigenesis and tumor progression. Diabetes mellitus might influence survival of CRC patients due to insulin-stimulated growth of colorectal cancer cells or inadequate treatment of persons with concomitant disease. However, it is unclear whether colorectal cancer patients with DM are more likely to receive a worse colorectal cancer prognosis compared to patients without DM. A meta-analysis has reported the effect of DM on CRC prognosis[5], but since 2013, a series of large-scale long-term cohort studies had comprehensively reported the outcome effect estimates on the relationship between DM and CRC prognosis, and their results were still consistent[6–20]. For example, in overall survival (OS) of CRC, several studies found that DM showed a significant decreased risk in OS[6, 7, 12–14, 17], and others found no link[8–11, 15, 16, 18–20]. The data from these studies has also allowed us to evaluate the relationship between DM and CRC prognosis more accurately. Thus we want to perform a meta-analysis to determine the relationship between DM and CRC prognosis, and provide a theoretical basis for clinical research. Our meta-analysis first reported the 5-year survival estimates on the effect of DM on CRC prognosis, and respectively analyzed the effects of DM on the colorectal, colon and rectal cancer from OS, cancer-specific survival (CSS), cardiovascular disease—specific survival (CVDS), disease-free survival (DFS), or recurrence-free survival (RFS).

## Methods

### Literature search

A systematic literature review was independently carried out by two groups (Bo Zhu, Bo Wu as a group, and Lu Zhang, Lixuan Wei as another group) in multiple databases (Pubmed, Web of Science, Embase and Google Scholar) up to March 19, 2017. In order to collect as many relevant studies as possible, we set the following search terms: (diabetes OR hyperglycemia OR glucose intolerance) AND (colorectal cancer OR colorectal neoplasms OR colon cancer OR colonic neoplasms OR rectal cancer OR rectal neoplasms) AND (prognosis OR survival analysis OR survival OR survival rate OR mortality). The reviewed reference lists from all the relevant original research and reviews were also searched to identify additional potentially eligible studies. There were no language or other restrictions. All retrieved studies were initially selected by reading the title and abstract. S1 File showed the detailed methods used for searching all the databases.

### Inclusion and exclusion criteria

The final included studies were identified by reading the full text, according to the inclusion and exclusion criteria. Three authors (Bo Zhu, Xiaomei Wu and Bo Wu) participated in this process, and any disagreements were solved by discussion.

The included studies in our meta-analysis should meet the following criteria: the study should (1) investigate the relationship between DM and CRC prognosis; (2) be cohort study; (3) provide the hazard ration (HR) or rate, which reflected overall survival (OS), cancer-specific survival (CSS), cardiovascular disease—specific survival (CVDS), disease-free survival (DFS), or recurrence-free survival (RFS); (4) provide the relevant data to calculate the corresponding outcome effect estimates.

The diagnostic criterion for DM and hyperglycemia was used by the World Health Organization (WHO) 1999 criteria or American Diabetes Association (ADA) 2010 guidelines. OS was defined as the time from the date of surgery to death from any cause. CSS was defined as the time from the date of surgery to death from colorectal cancer-specific cause of death. CVDS defined as the time from the date of surgery to death from cardiovascular disease -specific cause of death. DFS was defined as time from the date of surgery to tumor recurrence or occurrence of a new primary colorectal tumor or death from any cause. RFS was defined as the time from the surgery to tumor recurrence or occurrence of a new primary colon tumor[8, 21].

The exclusion criteria of our meta-analysis are: (1) the study did not investigate the relationship between the relationship between DM and CRC prognosis; (2) the study did not provide the relevant data to calculate outcome effect estimates (including HR and/or rate), which reflected OS, CSS, CVDS, DFS, or RFS; (3) the type of study excluded animal experiment, chemistry and cell-line research, letters to the editor, meetings abstracts, communications or review.

### Data extraction and conversion

The data from the final included studies were extracted independently by two authors (Bo Zhu and Xiaomei Wu). These authors used the standard table to extract the information, which included author, year of publication, country, type of study, sample size, population source, recruitment time, age, gender, patients with DM, DM ascertainment, type of cancer, outcomes, and adjusted variables. If the study provided more than two outcome effect estimates adjusted for different numbers of potential confounders, we extracted the estimate that adjusted for the highest number of potential confounders for analysis. If more than two studies provided the outcome effect estimates from the same population, we extracted the latest or highest-quality outcome effect estimates.

### Quality assessment

Two authors (Bo Zhu and Xiaomei Wu) independently conducted the quality assessment of the final studies included by using the Newcastle-Ottawa Quality Assessment Scale (NOS)[22]. The NOS is a semi quantitative method for assessing the quality of studies, and consisted of three main parts: selection (4 points), comparability (2 points) and outcome (3 points). Thus, the quality of study was determined on a scale from zero to nine points. Studies with seven or more points were regarded as “high quality”, studies with the points from four to six were regard as “moderate quality”, and otherwise, the study was regarded as “low quality”[23].

### Statistical analysis

The Stata v.12.0 software was used to conduct our meta-analysis and used the pooled outcome effect estimates and corresponding 95% confidence interval (CI) for OS, CSS, CVDS, DFS or RFS to analyze the relationship between DM and CRC prognosis. If the study did not provide the corresponding results, we used the Engauge Digitizer v.4.1 software (http://digitizer.sourceforge.net/) to extract survival rates from the Kaplan-Meier curves [24–26], the survival rates were entered in the spreadsheet by the method in Tierney’s article[24]. The process of extracting survival rates was performed by two independent authors (Dan Pei and Lixuan Wei) to make the extracted data more accurate. The heterogeneity in the included studies was evaluated by the Chi-square-based Q-test and I^2^ (I^2^ = 0% to 25%, no heterogeneity; I^2^ = 25% to 50%, moderate heterogeneity; I^2^ = 50% to 75%, high heterogeneity; I^2^ = 75% to 100%, extreme heterogeneity). When I^2^ was larger than 50%, a random effects model was used; otherwise, the fixed effects model was used.

We used subgroup analysis by region, type of study, sample size, population source and DM ascertainment to find the potential heterogeneity among the included studies. If the number of study was less than or equal to 1, we did not carry out the subgroup analysis. We used the sensitivity analysis to evaluate the robustness of the results by excluding each study in turn and obtaining the pooled estimates from the remaining studies. The purpose of sensitivity analysis was to evaluate the effect of a single study on the overall pooled estimates. If the number of study was less than or equal to 1, we did not carry out the subgroup analysis and sensitivity analysis.The possibility of publication bias was assessed using Begger's and Egger's test. Where publication bias existed, we also performed the Duval and Tweedie nonparametric “trim and fill” procedure to further assess the possible effect of publication bias in our meta-analysis. If the number of study was less than or equal to 2, we did not carry out the sensitivity analysis and publication bias test. A two-sided P value <0.05 in statistical process was considered significantly different.

## Results

### Search results

**Fig 1:**
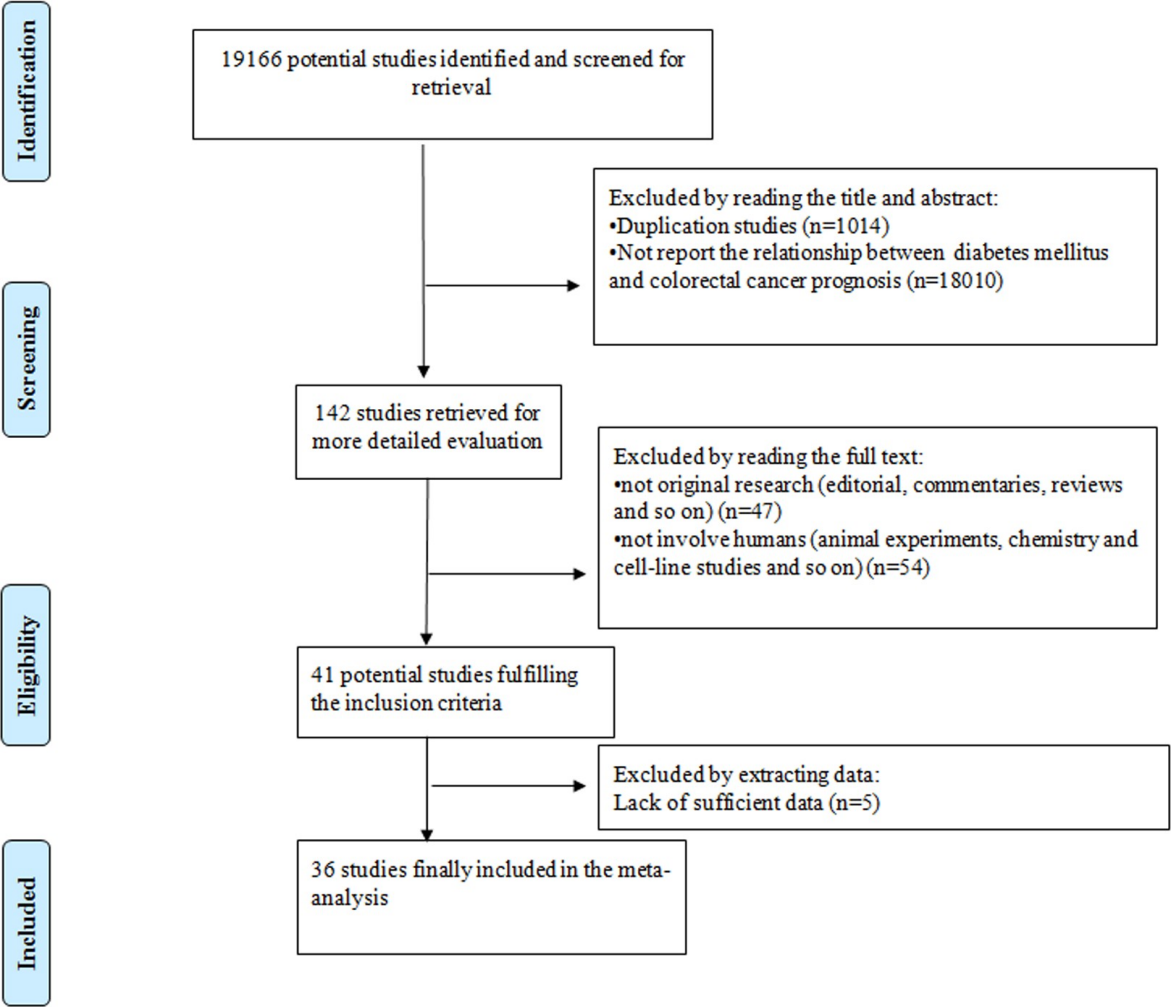
The study selection process for inclusion in our meta-analysis

Originally, we retrieved 19166 potential studies from four electronic databases. By reading the title and abstract, we found that 1014 studies were repetitive and 18010 studies did not report the relationship between DM and CRC Prognosis. By reading the full text, 101 studies were excluded for different reasons, and 5 studies did not provide sufficient data to calculate the outcome effect estimates. Finally, 36 studies were included in our meta-analysis[6–20, 27–47]. The study selection process for inclusion in our meta-analysis was shown in Fig 1.

### Study characteristics and quality

In our meta-analysis, year of publication ranged from 2003 to 2016, and the regions included 2 American countries[7, 13–15, 18, 19, 27, 30, 33, 37, 41, 42, 45, 46], 6 European countries[6, 11, 17, 28, 32, 39, 40, 44], 2 Asian countries[8, 9, 12, 16, 20, 29, 34–36, 38, 43, 47] and 1 Oceania country[31]; the included studies contained 15 retrospective[9, 10, 14, 16–20, 27, 33, 36, 37, 39, 41, 47] and 21 prospective[6–8, 11–13, 15, 28–32, 34, 35, 38, 40, 42–46] cohort studies; the sample size ranged from 391 to 1056243, and the mean age of study ranged from 46.4 to 72.07. In DM ascertainment, 25 studies[6, 8, 9, 11–15, 18, 19, 28, 29, 31, 33–37, 39–42, 44–46] used the method of medical records, 5 studies[16, 20, 38, 43, 47] used the method of blood sugar test, and 6 studies[7, 10, 17, 27, 30, 32] used the method of self-reported. To avoid the effects of confounders, we preferred to extract the adjusted outcome effect estimates, but we still found that the outcome effect estimates of 4 studies were not adjusted.

The quality score ranged from 5 to 9. 11 studies were evaluated as 9 scores, 7 studies were evaluated as 8 scores, 12 studies were evaluated as 7 scores, 4 studies were evaluated as 6 scores, and 2 studies were evaluated as 5 scores. All the included studies were regarded as moderate and high quality.

The characteristic and quality of the included studies is shown in Table 1.

**Table 1:** The characteristic and quality of the included studies.

**Author**	**Year**	Region	Type of Study	Sample Size	Population source	Recruitment time	Age (Year)	Gender (male/female)	Patients with DM (n)	DM ascertainment	Type of cancer	Outcomes	Adjusted variable	NOS score
Lee, S. J.	2016	Korea	retrospective	741	Hospital-based	1999–2010	65.20	440/301	634	Blood glucose test	colon cancer	adjusted HROS; 5-year OS	age and sex, WBC, CRP, total cholesterol, high density lipoprotein, low density lipoprotein, triglycerides	9
Paulus, J. K.	2016	USA	retrospective	21292	population-based	2001–2008	69.16	20866/426	4983	Medical records	colorectal cancer	adjusted HROS; 5-year OS	age, race, AJCC stage, BMI, co-morbidity index, CRC treatment, smoking status	8
Fransgaard, T.	2016	Denmark	retrospective	29353	Hospital-based	2003–2012	70.05	15495/13858	3250	Self-reported	colorectal cancer	adjusted HROS	age, gender, ASA score, BMI, blood transfusions, smoking, alcohol consumption, elective or emergency surgery, AL, type of cancer (colon or rectal) and year of operation	9
Yang, I. P.	2016	Chinese Taiwan	retrospective	520	Hospital-based	2005–2011	64.56	310/210	135	Blood glucose test	colorectal cancer	adjusted HROS and DFS	age, gender, stage, tumor size, location, invasive depth, vascular invasion, perineural invasion and serum blood sugar of CRC patients	9
Ramjeesingh, R.	2016	Canada	retrospective	1304	Hospital-based	2005–2011	71.09	764/540	277	Medical records	colorectal cancer	adjusted HROS; 5-year OS	age, gender, co-morbidities (cardiac, diabetic, renal,and respiratory), diabetes treatments (metformin or not), BMI, smoking history, alcohol history, family history of CRC, location of cancer (rectal vs. colon), stage at diagnosis and differentiation	9
Cui, G.	2015	China	retrospective	391	Hospital-based	2008–2013	—	222/169	58	Blood glucose test	colorectal cancer	unadjusted HROS; 5-year OS	—	5
Chen, K. H.	2014	Chinese Taiwan	Prospective	6937	Population-based	2004–2008	67.3	3946/2991	1371	Medical records	colon cancer	adjusted HROS and HRCSS; 5-year OS and 5-year CSS	age, gender, tumor stage, treatment, cirrhosis, and all other co-morbidities	8
Luo, J.	2014	USA	Prospective	46400	Population-based	2003–2009	>65	20638/25762	14813	Medical records	colorectal cancer; colon and rectal cancer	adjusted HROS, HRCSS, and HRCVDS; 5-year OS and 5-year CSS	age at diagnosis, gender, race, marital status, grade, census tract median income and co-morbidity	8
Waheed, S.	2014	USA	Prospective	16977	Population-based	2000–2005	>67	7094/9883	4414	Medical records	colorectal cancer	unadjusted HROS, HRCSS and HRCVDS; 5-year OS, 5-year CSS and 5-year CVDS	—	6
Tong, L.	2014	USA	retrospective	375462	Population-based	1975–2009	—	190189/185273	—	Medical records	colorectal cancer	adjusted HROS	age, gender, race, and regions	6
Walker, J. J.	2013	Scotland	Prospective	19505	Population-based	2000–2007	—	10417/9088	2387	Medical records	colon and rectal cancer	adjusted HROS	age, SES, stage and treatment	7
Bella, F.	2013	Italy	Prospective	1039	Hospital-based	2003–2005	—	593/446	373	Medical records	colorectal cancer; colon and rectal cancer	adjusted HROS and HRCSS; 5-year OS and 5-year CSS	age, gender, stage, type of treatment, morphology and grade	7
Jeon, J. Y.	2013	Korea	Prospective	4131	Hospital-based	1995–2007	59	2479/1652	517	Medical records	colorectal cancer; colon and rectal cancer	adjusted HROS, HRRFS, HRDFS and HRCSS; 5-year DFS	age, gender, BMI, family history of CRC, TNM stage, adjuvant therapy and the year of surgery.	9
Morrison, D. S.	2013	Asia Pacific region	retrospective	600427	Population-based	1961–1999	46.4	216154/384273	182569	Self-reported	Colorectal cancer, rectal and colon cancer	adjusted HROS	age, BMI, physical activity, height, drink, smoke, cholesterol, diabetes and education	9
Liu, D.	2013	China	retrospective	525	Hospital-based	2004–2011	63.2	310/215	86	Medical records	colorectal cancer	unadjusted HROS and HRDFS; 5-year OS and 5-year DFS	—	6
Cossor, F. I.	2013	USA	Prospective	2066	Population-based	1993–1998	71.92	0/2066	212	Self-reported	colorectal cancer	adjusted HROS and HRCSS; 5-year OS and 5-year CSS	age and stage at diagnosis	7
Huang, C. W.	2012	Chinese Taiwan	Prospective	1197	Hospital-based	2002–2008	64.18	673/524	283	Medical records	colorectal cancer	adjusted HROS and HRCSS; 5-year OS and 5-year CSS	age, gender, location, tumor size, BMI, albumin, histology, AJCC stage, Pre-op CEA, Post-op CEA, vascular invasion and perineurial invasion	8
Dehal, A. N.	2012	USA	Prospective	2278	Population-based	1992–1993	—	—	393	Self-reported	colorectal cancer	adjusted HROS, HRCVDS and HRCSS; 5-year OS, 5-year CSS and 5-year CVDS	gender, age at CRC diagnosis, BMI, smoking status, physical activity, red meat intake, and surveillance, epidemiology, and end results summary stage	6
van de Poll-Franse, L. V.	2012	Netherlands	Prospective	10862	Hospital-based	1997–2007	68.34	5806/5056	1224	Medical records	colon cancer	adjusted HROS, HRCSS; 5-year OS and 5-year CSS	age at diagnosis, gender, stage, number of examined lymph nodes, adjuvant therapy, SES, year of diagnosis, hypertension, CVD, cerebrovascular disease, previous cancer and lung disease	9
Yeh, H. C.	2012	USA	retrospective	18240	Population-based	1989	51.8	7795/10445	599	Self-reported	colorectal cancer	adjusted HROS	age, the square of age, gender, BMI, smoking, education level, hypertension treatment, and high cholesterol treatment	9
Morrison, D. S.	2011	UK	Prospective	17949	Population-based	1967–1970	—	17949/0	236	Self-reported	colon and rectal cancer	adjusted HROS	age at risk, height, BMI, plasma cholesterol, diastolic blood pressure, systolic blood pressure, physical activity, socioeconomic position and smoking	7
Huang, Y. C.	2011	Chinese Taiwan	Prospective	2762	Hospital-based	1998.1–2008.1	—	1756/1006	469	Medical records	colon cancer	adjusted HROS and HRCSS; 5-year OS and 5-year CSS	age, gender, stage, bowel perforation at diagnosis, bowel obstruction at diagnosis, poorly differentiated or undifferentiated histology	7
Lai, C. C.	2011	Korea	Prospective	2529	Hospital-based	1995–2008	—	1315/1214	307	Medical records	colon cancer	adjusted HROS; 5-year OS	age, gender, hypertension, cardiac disease, old CVA, liver cirrhosis, other disease, CEA level, albumin level, morbidity, tumorphology, histologic type, histologic grade and TNM stage	7
Sarfati, D.	2011	New Zealand	Prospective	11524	Hospital-based	1996–2003	—	5477/6047	1107	Medical records	colon cancer	adjusted HROS	age, gender, ethnicity, NZ deprivation quintiles and extent of disease;	7
Lieffers, J. R.	2011	Canada	retrospective	574	Population-based	2004–2006	64	335/239	72	Medical records	colorectal cancer	adjusted HROS	age, gender, stage, and all co-morbidities	8
Chiao, E. Y.	2010	USA	retrospective	470	Hospital-based	1999–2006	67.7	464/6	122	Medical records	colorectal cancer	adjusted HROS; 5-year OS	age, race, BMI, stage, treatment received and Deyo co-morbidity score	9
Chen, C. Q.	2010	China	Prospective	945	Hospital-based	1994–2002	62.3	556/389	26	Blood glucose test	colorectal cancer	adjusted HROS and HRDFS; 5-year OS and 5-year DFS	gender, surgery type, chemotherapy, TNM, gross type, differentiation, intestinal obstruction and location	8
Noh, G. Y.	2010	Korea	retrospective	657	Hospital-based	1997–2004	57.97	374/283	67	Medical records	colorectal cancer	adjusted HROS and HRRFS; 5-year OS and 5-year RFS	age, gender, BMI, stage, grade	7
Jullumstro, E.	2009	Norway	retrospective	1194	Hospital-based	1980–2004	72.07	628/566	97	Medical records	colorectal cancer	adjusted HROS and unadjusted HRCSS; 5-year OS and 5-year CSS	age, gender, cardiac disease, pulmonary disease, ASA, bowel obstruction, bowel perforation, location, stage, poor differentiation, mean percentage positive nodes after resection and adjuvant chemotherapy	9
van de Poll-Franse, L. V.	2007	Netherlands	Prospective	8328	Hospital-based	1995–2002	68.15	4465/3863	913	Medical records	colon and rectal cancer	adjusted HROS; 5-year OS	age, gender, stage, treatment and CVD	7
Shonka, N. A.	2006	USA	retrospective	1853	Hospital-based	1986–2003	—	891/962	255	Medical records	colon cancer	unadjusted HROS; 5-year OS	—	5
Polednak, A. P.	2006	USA	Prospective	9395	Population-based	1994–1999	—	4487/4908	1014	Medical records	colorectal cancer	adjusted HROS	age at diagnosis, gender, race, extent of disease at diagnosis, lymph-node status and poverty-rate category	7
Park, S. M.	2006	Korea	Prospective	14578	Population-based	1996–2004	50.8	14578/0	1223	Blood glucose test	colorectal cancer	adjusted HROS	age, alcohol consumption, BMI, fasting serum glucose level, cholesterol level, physical activity, food preference, blood pressure, and other co-morbidities (heart disease, liver disease, and cerebrovascular disease	8
Lemmens, V. E.	2005	Netherlands	Prospective	6931	Population-based	1995–2001	—	3660/3271	—	Medical records	colon and rectal cancer	adjusted HROS	age, gender, tumor stage, treatment and number of co-morbid conditions or single concomitant diseases	7
Coughlin, S. S.	2004	USA	Prospective	1056243	Population-based	1982	—	467922/588321	52803	Medical records	colon and rectal cancer	adjusted HROS	age, race, years of education, BMI, cigarette smoking history, alcohol consumption, total red meat consumption, consumption of citrus fruits and juices, consumption of vegetables, physical activity	7
Meyerhardt, J. A.	2003	USA	Prospective	3549	Hospital-based	1988–1992	61.92	2936/613	287	Medical records	colon cancer	adjusted HROS, HRRFS and unadjusted HRDFS; 5-year at OS, 5-year DFS and 5-year RFS	age, BMI, gender, race, baseline performance status, bowel obstruction, bowel perforation, stage of disease, presence of peritoneal implants, and completion of chemotherapy	9

DM: diabetes mellitus; HROS: HR on overall survival; HRCSS: HR on cancer-specific survival; HRCVDS: HR on cardiovascular disease specific survival; HRDFS: HR on disease-free survival; HRRFS: HR on recurrence-free survival; 5-year at OS: the 5-year overall survival rate; 5-year at CSS: the 5-year cancer-specific survival rate; 5-year at CVDS: the 5-year cardiovascular disease specific survival rate; 5-year at DFS: the 5-year disease-free survival rate; RFS: the 5-year recurrence-free survival rate; BMI: body mass index; AJCC stage: the American Joint Committee on Cancer; CRC: colorectal cancer; ASA score: American Society of Anesthesiologists Score; AL: anastomotic leakage; SES: the socioeconomic status; TNM: tumor-node-metastasis; CVD: cardiovascular disease; CVA: old cardiovascular accident; CEA: carcinoembryonic antigen; WBC: white blood cell; CRP: C-reactive protein.

### The pooled survival rate for the effect of DM on CRC prognosis

In colorectal cancer, the pooled 5-year OS rate in patients with DM was 49.8%, and that in patients without DM was 53.6%; the pooled 5-year CVDS rate in patients with DM was 90.5%, and that in patients without DM was 94.3%; the pooled 5-year CSS rate in patients with DM was 65.6%, and that in patients without DM was 69.0%; the pooled 5-year DFS rate in patients with DM was 60.9%, and that in patients without DM was 70.0%; the pooled 5-year RFS rate in patients with DM was 63.4%, and that in patients without DM was 68.5%. Similar results were also found in colon and rectal cancer. The detailed results on the pooled survival rate for the effect of DM on CRC Prognosis were shown in Table 2.

**Table 2:** The pooled survival rate for the effect of DM on CRC Prognosis.

	Colorectal cancer (%)	Colon cancer (%)	Rectal cancer (%)
OS			
Patients with DM	49.8 (45.9, 53.6)	49.9 (21.5, 78.2)	50.9 (46.0, 55.8)
Patients without DM	58.1 (53.5, 62.6)	56.5 (44.1, 68.9)	64.1 (62.0, 66.3)
CVDS			
Patients with DM	90.5 (85.9, 95.1)	—	—
Patients without DM	94.3 (89.1, 99.5)	—	—
CSS			
Patients with DM	65.6 (61.3, 69.8)	71.7 (55.1, 88.3)	67.0 (64.8, 69.2)
Patients without DM	69.0 (63.3, 74.7)	75.4 (59.4, 91.3)	74.8 (74.0, 75.7)
DFS			
Patients with DM	60.9 (46.2, 75.5)	59.3 (37.2, 81.5)	65.9 (63.0, 68.8)
Patients without DM	70.0 (56.8, 83.3)	69.5 (48.9, 90.1)	68.2 (67.2, 69.2)
RFS			
Patients with DM	63.4 (51.9, 74.9)	57.0 (51.3, 62.7)	—
Patients without DM	68.5 (64.8, 72.3)	65.0 (63.4, 66.6)	—

DM: diabetes mellitus; OS: overall survival; CSS: cancer-specific survival; CVDS: cardiovascular disease—specific survival; DFS: disease-free survival; RFS: recurrence-free survival.

### The overall pooled HRs for the effect of DM on CRC prognosis

In our meta-analysis, the number of studies on the colorectal cancer data provided was 23[6–10, 13–20, 27, 29, 30, 33, 36–39, 42, 43], the pooled HRs on OS and CVDS were statistically significant (HR on OS: 1.18, 95%CI: 1.12–1.24; HR on CVDS: 1.40, 95%CI: 1.29–1.52), the pooled HRs indicated that there were no significant difference on CSS, DFS and RFS. No publication bias was found in OS, CVDS, CSS and DFS.

The number of studies on the colon cancer data provided was 18[6, 8, 10–13, 28, 31, 40, 41, 44–47]. There was only one study on CVDS, and the pooled HR on CVDS was not analyzed. The pooled HRs on OS and DFS were statistically significant (HR on OS: 1.19, 95%CI: 1.10–1.27; HR on DFS: 1.35, 95%CI: 1.12–1.58), the pooled HRs indicated that there were no significant difference on CSS and RFS. Publication bias might exist in OS and CSS (OS: P for Begger test = 0.049, P for Egger test = 0.115; CSS: P for Begger test = 0.260, P for Egger test = 0.012), we used “trim and fill” analysis to deduce the potential unpublished studies, the results of OS and CSS(HR on OS: 1.19, 95%CI: 1.11–1.28; HR on CSS: 1.06, 95%CI: 0.98–1.14) were similar to the overall results, respectively.

The number of studies on the rectal cancer data provided was 10[6, 8, 10, 11, 13, 28, 40, 44, 45], there was only one study on CVDS, DFS and RFS, the pooled HRs on CVDS, DFS or RFS were not analyzed. The pooled HR on OS was statistically significant (HR on OS: 1.16, 95%CI: 1.04–1.29), the pooled HR indicated that there were no significant difference on CSS. No publication bias was found in OS and CSS.

The detailed results on the relationship between DM and CRC Prognosis are shown in Table 3.

**Table 3:** The overall pooled HR on the effect of DM on CRC Prognosis.

	Number of study	Model for meta-analysis	HR (95%CI)	I^2^ (%)	P for heterogeneity	P for Begger’s test	P for Egger’s test
**Colorectal cancer**							
OS	23[6–10, 13–20, 27, 29, 30, 33, 36–39, 42, 43]	R	**1.18(1.12, 1.24)**	64.8	<0.001	0.492	0.740
CVDS	3[13, 15, 30]	F	**1.40(1.29, 1.52)**	31.6	0.232	0.296	0.193
CSS	8[6–8, 13, 15, 29, 30, 39]	R	1.03(0.93, 1.12)	63.3	0.008	0.711	0.225
DFS	4[8, 9, 20, 38]	R	1.14(0.71, 1.58)	80.0	0.002	0.734	0.893
RFS	2[8, 36]	F	1.08(0.84, 1.23)	0.0	0.771	—	—
**Colon cancer**							
OS	18[6, 8, 10–13, 28, 31, 32, 34, 35, 40, 41, 44–47]	R	**1.19(1.10, 1.27)**	86.9	<0.001	0.049	0.115
CVDS	1[13]	—	1.35(1.26, 1.45)	—	—	—	—
CSS	6[6, 8, 12, 13, 28, 35]	F	1.07(0.98, 1.16)	38.9	0.146	0.260	0.012
DFS	2[8, 46]	F	**1.35(1.12, 1.58)**	0	0.447	—	—
RFS	2[8, 46]	F	**1.24(1.04, 1.44)**	0	0.634	—	—
**Rectal cancer**							
OS	10[6, 8, 10, 11, 13, 28, 40, 44, 45]	R	**1.16(1.04, 1.29)**	61.9	0.005	0.474	0.529
CVDS	1[13]	—	1.48(1.04, 1.29)	—	—	—	—
CSS	4[6, 8, 13, 28]	R	1.12(0.91, 1.32)	55.2	0.082	0.308	0.389
DFS	1[8]	—	0.98(0.76, 1.25)	—	—	—	—
RFS	1[8]	—	0.96(0.72, 1.28)	—	—	—	—

R: the random effects model; F: the fixed effects model; DM: diabetes mellitus; OS: overall survival; CSS: cancer-specific survival; CVDS: cardiovascular disease—specific survival; DFS: disease-free survival; RFS: recurrence-free survival.

### Subgroup analysis

Because of fewer studies on CVDS, CSS, DFS, and RFS, we used subgroup analysis on OS by the potential confounding factors, including region, type of study, sample size, population source, DM ascertainment, quality of studies and adjusted variables. In colorectal cancer, we found that the relationship between DM and CRC prognosis was significant in all groups, but not in Asian or blood glucose test groups. We found similar results in colon and rectal cancer. The detailed results on the subgroup analysis on OS for the effect of DM on CRC Prognosis were shown in Table 4.

**Table 4:** The subgroup analysis on OS for the effect of DM on CRC Prognosis.

	Colorectal cancer					Colon cancer					Rectal cancer				
	Number of study	Model for meta-analysis	HR (95%CI)	I^2^ (%)	P for heterogeneity	Number of study	Model for meta-analysis	HR (95%CI)	I^2^ (%)	P for heterogeneity	Number of study	Model for meta-analysis	HR (95%CI)	I^2^ (%)	P for heterogeneity
**Region**															
America	11[7, 13–15, 18, 19, 27, 30, 33, 37, 42]	R	**1.19(1.11, 1.27)**	78.0	<0.001	5[13, 41, 45, 46]	F	**1.21(1.14, 1.29)**	33.5	0.198	3[13, 45]	F	**1.16(1.01, 1.32)**	24.1	0.268
Europe	4[6, 16, 17, 39]	F	**1.25(1.12, 1.37)**	5.3	0.366	6[6, 11, 28, 32, 40, 44]	F	**1.16(1.09 1.24)**	1.7	0.406	5[6, 11, 28, 40, 44]	R	**1.26(1.03, 1.49)**	74.8	0.003
Asia	8[8–10, 20, 29, 36, 38, 43]	F	1.06(0.91, 1.22)	26.1	0.220	6[8, 10, 12, 34, 35, 47]	F	**1.25(1.12, 1.39)**	31.7	0.198	2[8, 10]	F	0.91(0.56, 1.25)	7.6	0.298
Oceania	0	—	—	—	—	1[31]	—	1.00(0.98, 1.02)	—	—	0	—	—	—	—
**Type of study**															
Retrospective	12[9, 10, 14, 16–20, 33, 36, 37, 39]	F	**1.14(1.09, 1.19)**	2.5	0.420	3[10, 41, 47]	F	0.98(0.72, 1.18)	0.0	0.416	1[10]	—	0.32(0.04, 2.39)	—	—
Prospective	11[6–8, 13, 15, 27, 29, 30, 38, 42, 43]	R	**1.22(1.12, 1.33)**	78.6	<0.001	15[6, 8, 11–13, 28, 31, 32, 34, 35, 40, 44–46]	R	**1.21(1.12, 1.29)**	89.0	<0.001	9[6, 8, 11, 13, 28, 40, 44, 45]	R	**1.17(1.05, 1.30)**	62.9	<0.001
**Sample size**															
≥ 10000	8[10, 13–15, 17, 18, 27, 43]	R	**1.14(1.07, 1.20)**	70.0	0.001	7[10, 11, 13, 31, 32, 45]	R	**1.14(1.01, 1.26)**	93.3	<0.001	4[10, 13, 45]	F	1.10(0.89, 1.32)	37.7	0.186
<10000	15[6–9, 16, 19, 20, 29, 30, 33, 36–39, 42]	R	**1.21(1.08, 1.33)**	55.6	0.005	11[6, 8, 12, 28, 34, 35, 40, 41, 44, 46, 47]	F	**1.22(1.13, 1.31)**	45.2	0.051	6[6, 8, 11, 28, 40, 44]	R	**1.21(1.01, 1.41)**	72.5	0.003
**Population source**															
Population-based	10[7, 13–15, 18, 27, 30, 33, 42, 43]	R	**1.20(1.12, 1.28)**	79.8	<0.001	8[10–13, 32, 44, 45]	F	**1.20(1.17, 1.23)**	0.0	0.456	6[10, 11, 13, 44, 45]	F	1.09(0.95, 1.23)	49.1	<0.001
Hospital-based	13[6, 8–10, 16, 17, 19, 20, 29, 36–39]	F	**1.14(1.02, 1.25)**	31.8	0.129	10[6, 8, 28, 31, 34, 35, 40, 41, 46, 47]	R	**1.18(1.06, 1.30)**	80.7	<0.001	4[6, 8, 28, 40]	R	**1.30(1.02, 1.58)**	75.2	0.007
**DM ascertainment**															
Medical records	14[6, 8, 9, 13–15, 18, 19, 29, 33, 36, 37, 39, 42]	R	**1.18(1.11, 1.24)**	70.7	<0.001	15[6, 8, 11–13, 28, 31, 34, 35, 40, 41, 44–46]	R	**1.20(1.11, 1.28)**	89.0	<0.001	9[6, 8, 11, 13, 28, 40, 44, 45]	R	**1.17(1.05, 1.30)**	62.9	0.006
Self-reported	5[7, 10, 17, 27, 30]	F	**1.29(1.08, 1.51)**	49.5	0.095	2[10, 32]	F	1.02(0.30, 1.73)	0.0	0.504	1[10]	—	0.32(0.04, 2.39)	—	—
Blood glucose test	4[16, 20, 38, 43]	F	0.95(0.65, 1.25)	27.2	0.249	1[47]	—	0.57(0.22, 1.47)	—	—	0	—	—	—	—
**Quality of studies**															
Moderate	5[9, 14–16, 30]	R	**1.16(1.03, 1.28)**	75.5	0.003	1[41]	—	1.00(0.77, 1.30)	—	—	0	—	—	—	—
High	18[6–8, 10, 13, 17–20, 27, 29, 33, 36–39, 42, 43]	R	**1.19(1.11, 1.27)**	47.4	0.014	17[6, 8, 10–13, 28, 31, 32, 34, 35, 40, 44–47]	R	**1.19(1.11, 1.28)**	87.6	<0.001	10[6, 8, 10, 11, 13, 28, 40, 44, 45]	R	**1.16(1.04, 1.29)**	61.9	0.005
**Adjusted variables**															
no	3[9, 15, 16]	F	1.03(0.96, .10)	0.0	0.823	1[41]	—	1.00(0.77, 1.30)	—	—	0	—	—	—	—
yes	20[6–8, 10, 13, 14, 17–20, 27, 29, 30, 33, 36–39, 42, 43]	R	**1.20(1.14, 1.26)**	57.8	0.001	17[6, 8, 10–13, 28, 31, 32, 34, 35, 40, 44–47]	R	**1.19(1.11, 1.28)**	87.6	<0.001	0	10[6, 8, 10, 11, 13, 28, 40, 44, 45]	R	**1.16(1.04, 1.29)**	61.9

R: the random effects model; F: the fixed effects model; DM: diabetes mellitus; OS: overall survival; CSS: cancer-specific survival; CVDS: cardiovascular disease—specific survival; DFS: disease-free survival; RFS: recurrence-free survival.

### Sensitivity analysis

The pooled HRs and their 95%CIs of sensitivity analysis were calculated by excluding one study at a time in colorectal cancer, colon cancer and rectal cancer, and the results indicated that the overall result was dependable. The results of sensitivity analysis were shown in Table 5.

**Table 5:** The sensitivity analysis of the overall pooled HR on the effect of DM on CRC Prognosis.

	The lowest HR (95%CI)	The highest HR (95%CI)
**Colorectal cancer**		
OS	1.18(1.12, 1.24)	1.38(1.31, 1.46)
CVDS	1.38(1.31, 1.46)	1.66(1.11, 2.51)
CSS	1.00(0.92, 1.09)	1.11(0.97, 1.27)
DFS	1.03(0.68, 1.58)	1.37(1.03, 1.83)
**Colon cancer**		
OS	1.18(1.10, 1.27)	1.22(1.17, 1.26)
CSS	1.03(0.97, 1.11)	1.13(1.04, 1.23)
**Rectal cancer**		
OS	1.15(1.02, 1.28)	1.22(1.09, 1.38)
CSS	1.08(0.91, 1.29)	1.24(0.93, 1.67)

DM: diabetes mellitus; OS: overall survival; CSS: cancer-specific survival; CVDS: cardiovascular disease—specific survival; DFS: disease-free survival; RFS: recurrence-free survival.

## Discussion

Our meta-analysis first analyzed both the 5-year survival rate and survival risk, which reflected the effect of DM on CRC prognosis. The results indicated that compared to patients without DM, patients with DM will have a 5-year shorter survival rate in colorectal, colon and rectal cancer, showed 18%, 19% and 16% decreased in OS, respectively. We also found similar results in CVDS, CSS, DFS and RFS. Due to the heterogeneity, we performed the subgroup analysis and sensitivity analysis to find the source of heterogeneity and make our results robust and credible. In subgroup analysis, though few results showed no statistical significance, we found that the results of subgroup analysis were generally similar to the overall results. When we carried out subgroup analysis by region, in Europe, patients with DM significantly have shorter OS in colorectal cancer, colon cancer and rectal cancer. In Asia, patients with DM significantly have shorter OS in colon cancer; there was no significance in colorectal cancer and rectal cancer, this may be the small sample size due to subgroup analysis. When we carried out subgroup analysis by type of study, there were significant differences in the results, except for that in prospective studies of colon cancer. When we carried out subgroup analysis by sample size and population source, the subgroup results were consistent with the overall results in colorectal and colon cancer, the results in size ≥ 10000 and population-based group did not show statistical significant in rectal cancer. When we carried out subgroup analysis by DM ascertainment, the results were consistent with the overall results in the group of medical records, except for that in the group of self-reported and blood glucose test. The sensitivity analysis also showed that the results of our meta-analysis were robust and credible.

Currently, the biological mechanism linkage between DM and CRC prognosis is still uncertain. This association may be mainly based on the effect of hyperinsulinemia, insulin resistance and cancer pathogenesis on the insulin/ insulin-like growth factor (IGF) system, which plays a critical role in the pathogenesis, progression, and prognosis of CRC. On the one hand, the insulin-like effects of IGF-1 interacting with associated receptors, such as IGF-1R, IR or hybrid receptors, play an important role in the maintenance of normal glucose homeostasis and etiopathogenesis of DM[48]. In DM patients, insulin resistance leads to a compensatory increase in insulin secretion, and by inhibition of IGF binding proteins, this hyperinsulinemia may increase the biological activity of IGF-1, which is an antiapoptotic and mitogenic factor[49]. On the other hand, insulin-like growth factors activate the IGF-1R, make it over expressed in cancer cells, and then trigger a number of intracellular signaling cascades that enhance cell cycle progression and inhibit apoptosis. Zhang et al indicated that IGF-1 and its receptor promoted both the growth and malignant transformation of adenomatous polyps[50]. Over expression of IGF-1, IGF-1R and IR were found in CRC group with DM than that in without DM[51]. The activation of insulin/IGF-dependent pathways has been also identified as a critical step contributing to several mechanisms of CRC resistance to both conventional and targeted therapeutic agents, leading to increased PI3K/Akt signaling that hinders the apoptotic signals triggered by chemotherapeutic drugs and desensitizes CRC cells to the effect of anti-EGFR antibodies[52]. Scartozzi et al. had reported that high IGF-1 expression correlated with poor clinical outcome in wild-type KRAS metastatic CRC patients treated with cetuximab and irinotecan. Their results indicated that engaging the IGF-1/IGF-1R system might enable tumor cells to escape anti-EGFR-mediated treatment as a consequence of IGF-1-driven stimulation of the PI3K–Akt pathway[53]. In recent years, some evidence suggested that IGF-1/IGF-1R polymorphisms are potential predictive/prognostic markers for cetuximab efficacy in metastatic CRC patients presenting wild- type KRAS[54].

In order to make our results more robust and credible, we made efforts in several ways. First of all, we not only searched the relevant studies in the four commonly used electronic databases, but also searched in Google Scholar, and tried our best not to miss the relevant studies. We also extracted the data on OS, CSS, CVDS, DFS and RFS, and used these indicators to evaluate the effect of DM on CRC prognosis. So far, our meta-analysis is the most comprehensive study of collecting indicators on the effect of DM on CRC prognosis. Second, we performed the quality assessment by NOS, which was widely used in meta-analysis and systematic reviews, and all the included studies were evaluated as high quality, which made our extracted data reliable. Third, we found that only one result in CSS of colon cancer existed publication bias, there were no publication bias in all other results. We used the “trim and fill” analysis to assess the possible effect of publication bias, but there was no significant change in the CSS result of colon cancer. The results of subgroup analysis and sensitivity analysis has also shown that our results were robust and credible. Finally, and most importantly, compared to previous studies[5], we not only routinely performed the pooled analysis on HR of OS, CSS, CVDS, DFS and RFS, which comprehensively reflect the difference of CRC prognosis between diabetic patients and nondiabetic patients; but also first extracted the 5-year survival rate from the included studies, and made the pooled analysis. Meanwhile, for collecting more useful data, we used the professional software to extract survival rate from the Kaplan-Meier curves[24, 25]. This would make the results stable, and give the researchers more intuitive impression on the effect of DM on prognosis in the fifth year.

There were several limitations in our meta-analysis. First, in order to collect the literatures more extensively, we searched the relevant articles in Google Scholar. If we found the relevant articles in Google Scholar, we purchased the article or sought help online[55].Second, in the included studies, we found that more studies focused on OS, compared to CSS, CVDS, DFS and RFS. In OS, the number of studies on colorectal, colon and rectal cancer was twenty-three, seventeen and ten. In CSS, CVDS, DFS and RFS, the maximum number of relevant studies was only eight. This might make the results unstable. In our meta-analysis, we analyzed both the 5-year survival rate and survival risk, and found their results were consistent. This indicated that our results were stable. Third, the results of our meta-analysis had a certain degree of heterogeneity. We performed subgroup analysis by the confounding factors, which might be the potential source of heterogeneity, and the results of subgroup analysis were similar to the overall results. We also performed the analysis of the effect of each study on the overall results sensitively, and did not find significant changes in the overall results.

In conclusion, our meta-analysis showed that DM could significantly decrease OS in CRC patients, but not CSS, CVDS, DFS and RFS. In future, to provide more evidence of clinical treatment, more high quality prospective cohort studies are needed to comprehensively analyze the effect of DM on CRC prognosis by CSS, CVDS, DFS and RFS.

## Supporting information

S1 PRISMA Checklist (DOCX)

S1 File The detailed methods used for searching all the databases. (DOCX)
