# Supplementary material for: The relationship between diabetes and colorectal cancer prognosis: A meta-analysis based on the cohort studies
Source: PLoS One. 2017 Apr 19;12(4):e0176068. doi: 10.1371/journal.pone.0176068 (PMC5397066; doi:10.1371/journal.pone.0176068)
Supplement: S1 File — (DOCX) [file pone.0176068.s002.docx]

**1. Search strategy for Pubmed**

**#1** ((diabetes [Text Word]) OR hyperglycemia [Text Word]) OR glucose intolerance [Text Word]

**#2** (((((colorectal cancer [Text Word]) OR colorectal neoplasm [Text Word]) OR colon cancer [Text Word]) OR colonic neoplasm [Text Word]) OR rectal cancer [Text Word]) OR rectal neoplasm [Text Word]

**#3** ((((prognosis[Text Word]) OR survival analysis[Text Word]) OR survival[Text Word]) OR survival rate[Text Word]) OR mortality[Text Word]

**#4** #1 AND #2 AND #3

**2. Search strategy for Web of Science**

**TOPIC:**(diabetes OR hyperglycemia OR “glucose intolerance”) *AND* **TOPIC:**(“colorectal cancer ” OR “ colorectal neoplasm ” OR “ colon cancer ” OR “ colonic neoplasm ” OR “ rectal cancer ” OR “ rectal neoplasm ”) *AND* **TOPIC:**(prognosis OR “survival analysis ” OR survival OR “ survival rate ” OR mortality)

**3. Search strategy for Embase**

**#1** ' diabetes '/exp OR ' hyperglycemia '/exp OR ' glucose intolerance '/exp

**#2** ' colorectal cancer '/exp OR ' colorectal neoplasm '/exp OR ' colon cancer '/exp OR ' colonic neoplasm '/exp OR ' rectal cancer '/exp OR ' rectal neoplasm '/exp

**#3** ' prognosis '/exp OR ' survival analysis '/exp OR ' survival '/exp OR ' survival rate '/exp OR ' mortality '/exp

**#4** #1 AND #2 AND #3

**4. Search strategy for Google Scholar**

(diabetes OR hyperglycemia OR “ glucose intolerance ”) AND (“colorectal cancer ” OR “ colorectal neoplasm ” OR “ colon cancer ” OR “ colonic neoplasm ” OR “ rectal cancer ” OR “ rectal neoplasm ”) AND (prognosis OR “survival analysis ” OR survival OR “ survival rate ” OR mortality)
